# Detection of Anthocyanins in Potatoes Using Micro-Hyperspectral Images Based on Convolutional Neural Networks

**DOI:** 10.3390/foods13132096

**Published:** 2024-07-01

**Authors:** Fuxiang Wang, Qiying Li, Weigang Deng, Chunguang Wang, Lei Han

**Affiliations:** 1 College of Mechanical and Electrical Engineering, Inner Mongolia Agricultural University, No.306 Zhaowuda Road, Hohhot 010010, China; wfx7888453@imau.edu.cn (F.W.); qy0412s@163.com (Q.L.); dengwg@imau.edu.cn (W.D.); hanlei@cug.edu.cn (L.H.); 2 Inner Mongolia Engineering Research Center of Intelligent Equipment for the Entire Process of Forage and Feed Production, Hohhot 010018, China

**Keywords:** microhyperspectral, anthocyanin content, colored potato, convolutional neural network, partial least squares regression

## Abstract

The color potato has the function of both a food and vegetable. The color potato not only contains various amino acids and trace elements needed by the human body but also contains anthocyanins. Anthocyanins have many functions, such as antioxidation, inflammation inhibition, vision improvement, and cancer prevention, so colored potatoes are deeply loved by consumers and have good market prospects. However, at present, the detection of anthocyanin content in color potatoes mainly depends on chemical methods, which are time-consuming and laborious, so it is necessary to study a fast and accurate detection method. In this study, microscopic hyperspectral equipment was used to collect the spectral information of the outer skin and inner skin of potatoes. The original spectrum, pretreatment spectrum, and characteristic spectrum variables of the outer skin and inner skin were predicted by the convolution neural network (CNN) algorithm and partial least squares regression (PLS) algorithm, respectively, and the performance of the model was evaluated by the prediction set correlation coefficient (Rp), prediction set root mean square error (RMSEP), correction set correlation coefficient (Rc), correction set root mean square error (RMSEC), and residual prediction deviation (RPD). The results revealed that the inner skin Raw + CNN model constructed under raw spectral data is optimal with Rc = 0.9508, RMSEC = 0.0374%, Rp = 0.9461, RMSEP = 0.2361% and RPD = 4.4933. The inner skin Savitzky-Golay (SG) + Detrend (DET) + CNN model constructed from pre-processed spectral data is optimal with Rc = 0.9499, RMSEC = 0.0359%, Rp = 0.9439, RMSEP = 0.2384%, RPD = 4.6516. The inner skin DET + competitive adaptive reweighted sampling (CARS) +CNN model constructed from the feature-based spectral data was optimal with Rc = 0.9527, RMSEC = 0.0708%, Rp = 0.9457, RMSEP = 0.2711%, and RPD = 4.1623. It can be seen that the Rp, RMSEP, Rc, RMSEC, and RPD values for modeling the spectral information of the inner skin were higher than those of the outer skin under the three different spectral data. The prediction accuracy of the model built by the CNN algorithm was better than the conventional algorithm PLS, the application of the CNN algorithm in inner skin can achieve accurate prediction of anthocyanin content in potato.

## 1. Introduction

Colorful potatoes are rich in flavonoids with antioxidant properties such as anthocyanins, which have bright colors and are responsible for the color of fruits, vegetables, flowers, and plants [1]. Studies have shown that anthocyanins not only impart bright colors to plants but also resist ultraviolet rays, diseases, insect pests, and low temperatures [2,3,4]. In terms of nutrition and health care, anthocyanins are natural and powerful free radical scavengers [5] that have various physiological functions such as reducing the growth rate of cancer cells, regulating blood sugar, and enhancing vision as well as anti-aging and anti-tumor properties. Therefore, eating colored potatoes is beneficial to human health [6,7,8]. However, there are few studies on the detection of anthocyanins in potatoes at present. Currently, research on potato anthocyanins has mainly focused on their function, accumulation mode, characteristics, and content using stoichiometric methods. For the detection of anthocyanins, the pH-differential method and different high-performance liquid chromatography (HPLC) methods in combination with a photodiode array detector or mass spectrometry (MS) are most frequently used in the food industry as well as in research [9]. However, the detection process is complicated and redundant, which significantly affects the accurate detection and quality classification of potato anthocyanins.

Some scholars have also conducted some research on the rapid detection of anthocyanins and have made some achievements. Huck C W et al. [9] used near-infrared spectroscopy to quickly detect the total anthocyanin content in Sambucus fruits, used a partial least squares regression (PLSR) model to predict, and found that using near-infrared spectroscopy is a reliable detection method that can realize the prediction of anthocyanin content. U. S. Dinish [10] used a new custom portable handheld Vis-NIR spectrometer that collected the reflectance spectra of red and green lettuce leaves, wirelessly transmitted the spectral data via Bluetooth, and provided the raw spectral data and processed information, after which he predicted the anthocyanin content of red and green lettuce and found a correlation coefficient of 0.84. It can be seen that the handheld spectrometer can accurately detect the content of anthocyanins in plants. However, these studies are all point measurement methods, and the detection area is small.

Only a small area near the probe can be detected, and the overall information of the tested sample cannot be obtained.

Microscopic hyperspectral imaging technology is a combination of atlas technology, integrating microscopic imaging and spectral information, which can not only obtain the microscopic cell morphology information of substances but also the corresponding spectral information. Currently, it is mainly used in the medical field and rarely in agriculture. It can enlarge information that is invisible to the naked eye to the required multiple under a microscope. By directly projecting the beam to the target area and narrowing the measurement range, spectral and spatial information of tissue slices can be obtained [11,12,13], thereby fundamentally improving the detection accuracy. Minghua [14] used microscopic hyperspectral imaging technology to detect POD, CAT, and SOD activity indices of tomato leaves under salt stress and integrated them with spectral image information to build a quantitative model of antioxidant enzymes in tomato leaves under salt stress, realizing the spectral stripping and visual distribution of single antioxidant enzymes in cells. Xuan et al. [15] studied the detection of chlamydia spores in contaminated soil, obtained the microscopic hyperspectral image of perispora chlamydia in contaminated soil, and established the detection model. Jianhong [16] studied the changes of antioxidant active molecules and malondialdehyde (MDA) in mutton at different storage periods at 4 °C and combined with 400–1000 nm micro-hyperspectral technology, conducted rapid detection and analysis of total superoxide dismutase (T-SOD) activity, catalase (CAT) activity and malondialdehyde content. The feasibility of simultaneous determination of T-SOD, CAT, and MDA in mutton by micro-hyperspectral imaging was verified by Jinping [17]. Relying on the early detection method involving the use of micro-high spectral imaging technology to detect mushroom wolf molds, a combination of the BS-NET-FC band selection algorithm and MTCEM to high-spectrum image detection thickness of the thick spore micro-spectrum image was proposed. Great spores reduce redundant band images while effectively detecting the spore target. Lu [18] used the spectral information of microscopic hyperspectral imaging technology to conduct rapid non-destructive testing research on soluble protein content, glutathione (GSH) content, peroxidase (POD) activity and catalase (CAT) activity of mutton and established models, and these models generated good results. Huan [19] used visible micro-hyperspectral technology to detect the activity of superoxide dismutase (SOD) in mutton muscle cells. The above-mentioned studies confirm that micro-hyperspectroscopy is feasible for internal quality detection.

The traditional regression detection algorithm used currently in the prediction process of potato internal components is not suitable for the detection of large sample sizes, whereas the deep learning algorithm can achieve linear and nonlinear calculations through convolution and activation functions to extract the linear and nonlinear features of samples. In addition, the deep learning model is highly robust and can withstand the continuous detection of a large sample size. Therefore, deep learning has gradually become widely used in the regression analysis of spectral data [20,21,22]. Convolutional neural networks (CNN), as classic deep learning neural networks, have also been successfully applied to one-, two-, and three-dimensional data analysis [23,24,25,26,27].

Therefore, a convolutional neural network model based on deep learning of an artificial intelligence algorithm was used in this study to predict anthocyanin content. The specific research objectives were as follows:

(1) To obtain spectral information on the microstructures of different parts of potatoes.
(2) To build a model based on the CNN, conforming to the characteristics of the spectral data.
(3) To construct a convolutional neural network and partial least regression prediction model of potato anthocyanins based on the original spectrum, pre-process the spectrum and characteristic spectrum variables and obtain the optimal prediction model through comparative analysis.

At present, no scholars have used micro-hyperspectral equipment and deep learning algorithms to detect anthocyanin content in potatoes. This study provides a reference for future research in related industries.

## 2. Materials and Methods

### 2.1. Preparation of Experimental Samples

Potatoes were purchased from a farmers’ market in Hohhot, Inner Mongolia Autonomous Region. Fresh round or oval potatoes, which are intact and with no decay or mechanical damage, little difference in shape, and belonging to red and black diamond varieties were selected. The potatoes were cleaned using water before the experiment and placed at the laboratory temperature for 8 h. In this study, freehand sections were selected, and rectangular blocks of 8 mm × 6 mm × 15 mm of uniform size were cut with a single knife blade from the outer skin and inner skin of potatoes, respectively. They were cut into uniform slices with a knife blade and then rinsed briefly under distilled water using tweezers. After rinsing, the surface moisture was absorbed with absorbent paper and placed on the slide, and the cover slide was used for simple sealing. The outer skin and inner skin of the potatoes constituted 576 samples, respectively.

### 2.2. Hyperspectral Image Acquisition

#### 2.2.1. Microscopic Hyperspectral Imaging System

**Figure 1:**
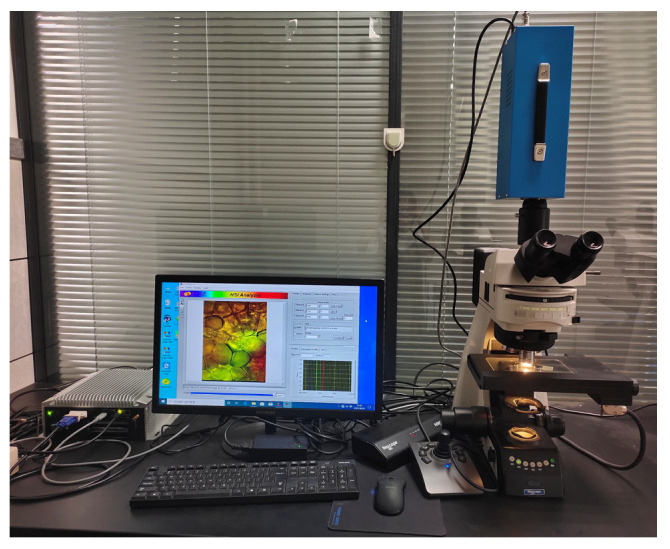
Micro-hyperspectral equipment.

In this study, a microscopic hyperspectral system was established by Wuling optics (Taiwan Province, China), as shown in Figure 1, which was composed of an optical microsystem, a spectral scanning imaging instrument, and a data acquisition system. An optical microscopy system primarily comprises a microscope and an electric carrier table. Multiples of 5, 10, 20, 50, and 100 times were obtainable for the objective mirrors, and a multiple of 10 was available for the eyepiece.

The interior of the spectral scanning imager is mainly composed of acousto-optic tunable filters (AOTF), their drivers, and CCD imaging equipment. Among them, the spectral scanner has a spectral range of 365–1025 nm, a total of 616 spectra, and a spectral resolution of 2.8 nm. The camera resolution of the imaging device is 1.4 million pixels, and the slit size is 30 µm.

#### 2.2.2. Micro-Hyperspectral Data Acquisition

Sample collection process:

(1) Before collecting the micro-high-resolution spectral images, the instrument and equipment were maintained open for 30 min to ensure that the light source irradiation intensity was stable. This study used the transmitted light source.
(2) The potato slices were fixed on a microscope carrier table.
(3) The image collection software Hyperspec was opened; the carrier table was initialized, and preliminary focusing was attained; the eye mirror adjustment focal length was observed to make the image clear; the image of the image was avoided; and the strength of the light source was adjusted until the sample reached a reasonable light source exposure value.
(4) The collection parameters were set in Hyperspec software. The specific parameters are listed in Table 1.
(5) The software was then executed, and the instrument was scanned until the image collection was completed. This process was repeated to collect all samples.

#### 2.2.3. Micro-Hyperspectral Image Correction

Owing to the inherent instability of the light source of the hyperspectral imaging system and the influence of external noise and the transmission process operation, the acquired hyperspectral images contain noise in certain bands. Therefore, before acquiring the micro-hyperspectral images, a lens cover and standard whiteboard were used to obtain all white and black images, respectively. Subsequently, in the acquisition software of the imaging system, the original image is corrected to black and white according to Equation (1) [19]:

(1) $$C=\frac{R-B}{W-B}\times 100\%$$

where C is the corrected image, R the original image, B the black reference image obtained by completely covering the camera lens with a lens cap (approximately 0% reflectivity), and W the white-calibrated image (approximately 100% reflectivity).

### 2.3. Experimental Methods

#### 2.3.1. Determination of Anthocyanin Content

The measurement of anthocyanins in potatoes was based on the Chinese agricultural industry standard “NY/T 2640-2014 Plant-Dedicated Flower Circin Primrose Method” [28]. The main principle was to extract anthocyanins from potatoes using ethanol and water and hydrolyze anthocyanins into anthocyanins by boiling water bath. The anthocyanins were determined using high-performance liquid chromatography, the retention time was qualitative, and the external standard method was quantitative.

#### 2.3.2. Extraction of Microscopic Hyperspectral Data

The corrected potato micro-hyperspectral images were imported into ENVI 5.3 software (ITT Visual Information Solutions, Boulder, CO, USA), and a Region of interest (ROI) area of 100 × 100 was selected on the complete cell structure with color using the square tool. The average spectral transmittance of the ROI was extracted from the spectral data of each sample. Using this method, the spectral information of the samples was successively extracted according to the sequence of labels, and the spectral data matrix of the outer and inner skin was established as 576 × 616 pixels.

#### 2.3.3. Micro-Hyperspectral Data Pre-Processing

Owing to the existence of mechanical noise and baseline drift in the original spectrum, it was necessary to conduct pre-processing to eliminate unnecessary information. In this study, the established data matrix was pre-processed, and the spectral pre-processing algorithms adopted were the standard normal variable transformation (SNV), Detrending method (DET), convolution smoothing (Savitzk-Golay), and their combinations, SG-SNV, SG-DET, and SNV-DET. SNV is the most widely used method in spectral data pre-processing, which is conducive to correcting changes in path length and spectral intensity and eliminating the interference of the nonlinear light scattering effect [29]. The SG can improve the smoothness of the spectrum and reduce noise interference. DET is a polynomial baseline correction method, which is used to eliminate baseline offset and curvature in spectral signals. It highlights the absorption peak of the spectral curve by subtracting an optimal trend line fitted by a polynomial from the original spectral curve [30]. DET has great advantages in eliminating the interference of spectral data by multicollinearity, baseline drift, and curvature. In this study, spectral data were pre-processed using the Unscrambler X 10.1 (CAMO AS, Oslo, Norway) software.

#### 2.3.4. Feature Wavelength Selection

High-spectrum data contain hundreds of thousands of continuous wavelengths with redundant and multi-common linearity. In this study, improved competitive adaptive weighted (CARS) and continuous projection algorithms (SPA) were used to eliminate redundant wavelengths and select the variables for the best performance.

The competitive adaptive reweighting algorithm (CARS) is an algorithm that Li et al. simplified and improved on the original CARS algorithm [31]. This method is based on Darwin’s evolutionary theory. In sample selection, adaptive reweighting sampling technology is used to select wavelength points with large absolute values of regression coefficients in the PLS model. Wavelength points with small weights are eliminated, and the subset with the smallest root-mean-square error RMSECV is selected by means of cross-validation, which is the optimal variable subset. In this study, 50 Monte Carlo samples and 10 cross-validations were used.

The SPA method is a forward variable-selection method that uses a simple projection operation to obtain a collinear minimum subset of variables. Therefore, the characteristic wavelength is extracted from the entire band, most of the redundant information in the original spectral matrix is eliminated, and the modeling conditions are improved. The basic principle of SPA is to project a set of wavelength subsets into a vector space and select the wavelength subset with the least redundancy [32]. The number of characteristic wavelengths was set in advance, and the parameters for the minimum and maximum numbers of variables selected in the SPA program were 1 and 30, respectively.

#### 2.3.5. Establishment of Regression Prediction Model

To compare and analyze the differences between the traditional regression algorithm and the popular deep learning algorithm in spectral information processing, the traditional linear partial least squares regression algorithm and convolutional neural network algorithm were selected in this study.

##### Partial Least Squares Regression Algorithm

Partial least squares regression (PLSR) [33,34] is the most widely used linear regression algorithm at present. Owing to its modeling principles and structure, this model is often the first choice for building predictive models. It has the advantage of considering both matrices X (spectral data) and Y (starch content). This can solve the problem of having a large number of variables or collinear variables in the original data. Partial least squares regression analysis was used to convert the original data into a limited number of independent latent variables (LVs). The optimal number of potential variables was determined by minimizing the sum of the squares of error (RMSE) to prevent model over-fitting or under-fitting. This is typically performed through cross-validation. In this study, the number of LVs was optimized using a 10-fold cross-validation method, the maximum number of LVs was set to 15, and the LVs value with a smaller error was selected according to the modeling effect. The ratio of the PLS model training set and test set was 2:1.

##### Convolutional Neural Network Regression Algorithm

As the convolutional neural network model can use a hierarchical structure, it has the characteristics of translation and other changes [35,36]. Thus, even if the appearance of the data changes, the original information of the data will not be lost, and effective information can still be identified and extracted. The convolutional neural network also has the feature of parameter sharing. The convolutional kernel moves in the same layer by means of translation and shares a set of convolutional kernel parameters when feature extraction is performed at each position. Therefore, the convolutional neural network significantly reduces the number of parameters, accelerates the learning rate, and prevents overfitting.

Combined with the characteristics of spectral data, this study proposes to further optimize the design of the basic convolutional neural network.

1. Combined with the spectral data and several experiments, three convolution layers were built, and the number of convolution nuclei in each layer was 16 and the sample ratio of the model training set and the test set is 4:1.
2. This study proposes introducing batch normalization (BN) after the convolutional layer, standardizing the data after convolution, and then inputting it into the next layer [37], which can significantly simplify the data after the convolutional layer and improve the speed after extracting useful information.
3. In this study, the rectified linear unit (ReLU) activation function was introduced after normalization.

##### Data Processing Software

The pre-processing method in this study used the Unscrambler software, and the PLS algorithm was implemented in MATLAB (R2019). The CNN program in this study is executed on the computer with the following specifications: Windows 10 system, Intel(R) Core (TM) i7-9700 CPU, 3.00 GHz, and memory 32 GB (Intel, Santa Clara, CA, USA). The CNN algorithm program is based on the Python language, using the PyTorch (Professional 2021) development environment. The learning rate was set to 0.001, and the number of training rounds was 100. The training hardware was an NVIDIA 2070 GPU for parallel computing.

## 3. Results and Discussion

### 3.1. Characteristic Analysis of Spectral Information

**Figure 2:**
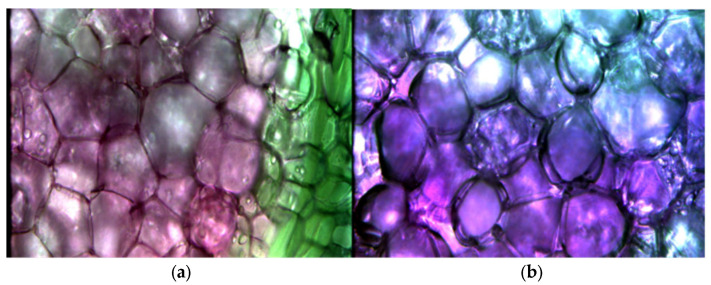
(**a**) Red Mei; (**b**) Black Jingang.

In this study, hyperspectral image information under the microscope of the outer and inner skin of two kinds of potatoes, Red Mei and Black Jingang, was collected, as shown in Figure 2.

**Figure 3:**
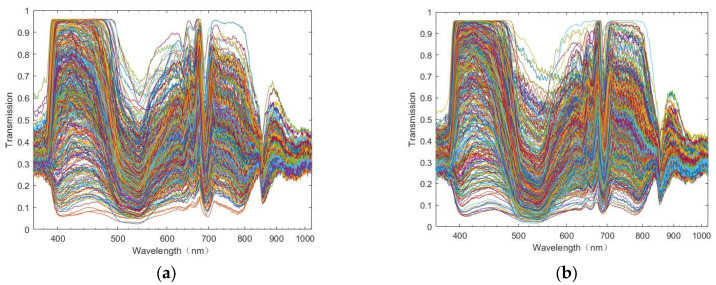
Raw spectrum: (**a**) Spectral information of the outer skin; (**b**) Spectral information of the inner skin.

As shown in Figure 3, the spectral information of the outer and inner skin shows that the trend of the raw spectral information of the two varieties is consistent, indicating that the spectral information under the microscope is not significantly affected by the variety. Further, by comparing the outer skin and the inner skin part, it was also found that the spectral trend of the inner and outer skin was consistent, which also showed that the spectral difference between the inner skin part and the outer skin was not large; however, whether there was a difference after the final application of algorithm modeling needs to be further studied.

Visible/near-infrared spectroscopy is based on the vibration of molecular bonds such as C-H, O-H, and N-H. Anthocyanins are flavonoid compounds with a C-skeleton of C6-C3-C6 as the basic structure, containing C-H, O-H, C-O, and other chemical bonds [38]. Therefore, visible/near-infrared spectroscopy can be used to predict anthocyanin content [39]. It can also be observed from the original spectrum in Figure 3 that there are evident trappings in the spectrum at approximately 530, 700, and 850 nm. The main pigment information was observed at approximately 530 and 700 nm, and anthocyanins were soluble pigments, which is consistent with previous studies [40,41]. The absorption peak at approximately 850 nm was due to the third overtone of C-H, which represents the absorption band of glucose and is related to the hydrocarbon group of C-H [42,43].

**Figure 4:**
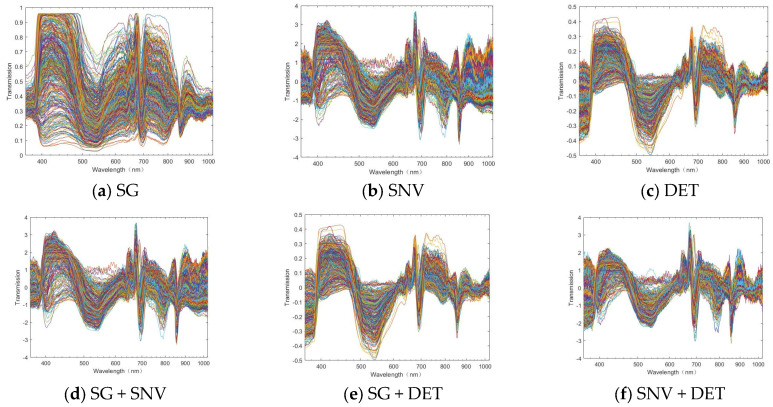
After the pre−processing of the outer skin.

**Figure 5:**
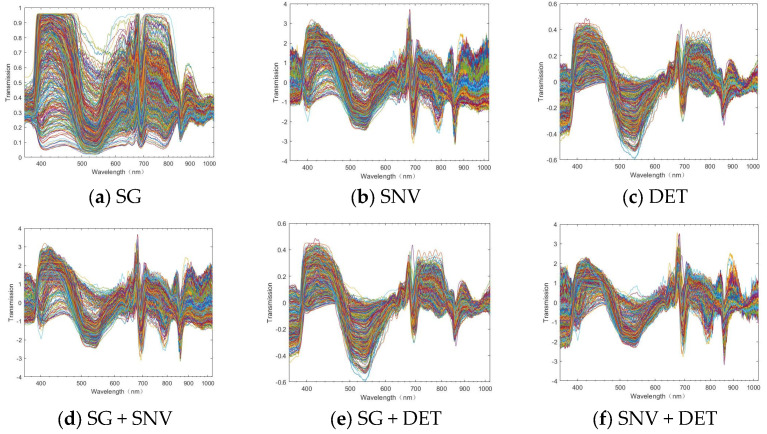
After the pre−processing of the inner skin.

It can be observed from the raw spectral data in Figure 3 that there is a certain raw edge, that is, noise, in the spectral information. Hence, it is necessary to pre-process the original data. SG smoothing, SNV, DET, SG + SNV, SG + DET, and SNV + DET were used to pre-process the original spectra of the inner skin part and the outer skin part, respectively, and the results are shown in Figure 4 and Figure 5. It can also be observed from the image that SG smoothing cannot generate evident changes from the original spectrum that are distinguishable with the naked eye. However, the spectral data value has changed, the other pre-processing methods generate clear changes, and the spectrum becomes more clustered and has fewer burrs.

### 3.2. Characteristic Wavelenght Extraction Analysis

In this study, feature wavelength extraction was performed on pre-processed data using CARS and SPA. The partial least squares regression and convolutional neural network algorithms were used to predict the internal anthocyanin content of potatoes in the spectra after feature extraction, and the results of the two algorithms were compared and analyzed. The characteristic wavelength variables extracted from the outer skin are shown in Table 2, and the characteristic wavelength variables extracted from the inner skin are shown in Table 3.

As shown in Table 2 and Table 3 for potato skin, the SG + SPA and SNV + DET + SPA algorithms extracted the least number of characteristic variables (12). Compared with the original spectral data, the number of input spectral variables was reduced by 98.05%. In the potato endothelium, the SNV + DET + SPA algorithm extracted the least number of characteristic variables (11), and the input spectral variables were reduced by 98.2% compared with the original spectral data. Studies have shown that the SPA algorithm extracts relatively few variables in the feature extraction of skin and inner skin parts, and this algorithm is more dominant in feature variable extraction because it identifies the least useful data, simplifies the input of prediction model data, avoids the input of redundant data, and plays a significant role in reducing the algorithm’s operation time.

### 3.3. Different Algorithms Predict Anthocyanin Content

#### 3.3.1. PLS Method Was Used to Predict Anthocyanin Content

In this study, the PLS method was used to predict the anthocyanin content in the original spectrum, pretreatment spectrum, and characteristic wavelength variable spectrum of two types of red, black, and gold potatoes. The results are presented in Table 4 and Table 5.

It can be observed from Table 4 that the PLS anthocyanin predictive model was established based on the original spectrum, RC = 0.7874, RMSEC = 0.429%, RPD = 2.30, and RPD value greater than 2, indicating that this model can predict the content of anthocyanins. It can also be noted that the PLS model based on the original spectrum-based construction is better than using some pre-processing and feature wavelength variables. Pre-processing and feature wavelength extraction methods cannot accurately remove and extract the main variables from the original spectral data. However, there are too many models based on the original spectrum construction, and redundant information exists between the spectrum bands. The performance of the PLS predictive model built using the original spectrum was low. Therefore, it is necessary to find the best pre-processing and feature variable method to reduce complexity and simplify the model.

According to the results in Table 4, for the PLS anthocyanin prediction model built based on the pretreatment method, the model with the best effect was SNV + PLS (Rp = 0.7496, RMSEP = 0.435%, Rc = 0.7439, RMSEC = 0.449%, RPD = 2.25, and RPD value greater than 2). This model can be used to predict potato anthocyanins quantitatively. The optimal PLS anthocyanin prediction model based on the characteristic wavelength was DET + CARS + PLS, with Rp = 0.8593, RMSEP = 0.37%, Rc = 0.8158, RMSEC = 0.334%, RPD = 2.64, and an RPD value greater than 2.5. This shows that the model can predict the anthocyanin content in a stable and accurate manner.

Table 5 presents the results of the PLS model constructed for inner skin cells. The results showed that the PLS anthocyanin prediction model established based on the original spectral information was ideal (RPD = 2.45), indicating that the model can accurately predict anthocyanins. However, owing to excessive input data and the addition of redundant information between the spectral data, the operational efficiency of the model is low. Therefore, it is necessary to optimize the pre-processing and feature wavelength extraction algorithms to reduce the complexity and simplify the model.

As shown in Table 5, the optimal PLS anthocyanin prediction model built based on the pretreatment method was SNV + PLS, with Rp = 0.839, RMSEP = 0.395%, Rc = 0.7707, RMSEC = 0.446%, and RPD = 2.50. According to the model evaluation criteria, this model could completely predict the anthocyanin content of potatoes. The optimal PLS anthocyanin prediction model based on the characteristic wavelength was the DET + CARS + PLS model, with Rp = 0.9287, RMSEP = 0.325%, Rc = 0.9227, RMSEC = 0.329%, RPD = 3.03, and an RPD value greater than 2.5. This shows that this model has strong robustness and stability and can accurately predict anthocyanins.

From Table 4 and Table 5, it can be observed that SNV + PLS is the ideal model for pre-processing, DET + CARS + PLS is the optimal model for feature variables, and DET + CARS + PLS is the best model among all models, indicating that the DET pre-processing algorithm can effectively reduce noise and remove unwanted information. The CARS algorithm can extract more effective feature variables, and the PLS algorithm can accurately predict the anthocyanin content in potatoes.

It can be noted from the results in Table 4 and Table 5 that under the premise of using the same algorithm, the models established for the outer and inner skin have different effects, indicating that the sampling location still affects the prediction accuracy. Further, the prediction accuracy of the models constructed for the inner skin was better than that of the outer skin, indicating that the spectrum of the inner skin is most closely related to the anthocyanin content. If a conventional algorithm is used to predict the anthocyanin content of potatoes, then the endothelium is the preferred location.

#### 3.3.2. Prediction of Anthocyanin Content by CNN Method

In this study, a CNN prediction model for anthocyanin content in potatoes was constructed based on the original full spectrum, pretreatment spectrum, and characteristic wavelength variable spectrum of potato skin and inner skin cells. The dataset was randomly divided into a training set (80%) and a test set (20%). The training set is used to train the model so that the model can learn features, and the test set is used to check the model effect.

The results of the CNN model constructed on the outer skin are listed in Table 6.

As shown in Table 6, the CNN model was built based on the original full spectrum, Rc = 0.9486, RMSEC = 0.0312%, Rp = 0.9446, RMSEP = 0.2291%, and RPD = 4.41, and the RPD value was much higher than 2.5, indicating that the CNN model built based on the original full spectrum was very robust. It can accurately predict anthocyanin levels.

SG, SNV, DET, and their combinations were used to preprocess the original spectrum of the outer skin to establish a CNN prediction model. The DET + CNN model had the best accuracy, with Rc = 0.9499, RMSEC = 0.0235%, Rp = 0.9457, and RMSEP = 0.2234%. RPD = 4.4893, and the RPD value of the model was much higher than 2.5, indicating that this model could accurately predict the level of anthocyanins.

CARS and SPA feature extraction algorithms were used to extract the feature wavelength variables. The DET + CARS + CNN model exhibited the best prediction accuracy: Rc = 0.9524, RMSEC = 0.0657%, Rp = 0.9468, RMSEP = 0.2651%, and RPD = 4.0942. The RPD value of the model is still greater than 2.5, and this model can achieve accurate prediction of anthocyanins. As shown in the above table, the accuracy of the CNN model built based on SPA feature extraction is relatively general. The feature information extracted by SPA and input spectral data are limited; therefore, the CNN model learns less effective information during the training process, whereas the characteristic wavelength information variables of anthocyanins extracted by SPA in this study are not sufficiently accurate resulting in average model accuracy.

**Figure 6:**
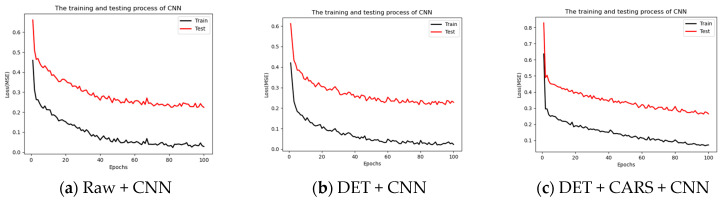
Results of the optimal outer skin model.

The training and testing processes of the Raw + CNN, DET + CNN, and DET + CARS + CNN models are shown in Figure 6.

The specific results of the CNN model constructed for inner skin cells are listed in Table 7.

As shown in Table 7, the Raw + CNN model built based on the original full spectrum had a high prediction accuracy: Rc = 0.9508, RMSEC = 0.0374%, Rp = 0.9461, RMSEP = 0.2361%, and RPD = 4.4933. The RPD value of this model was very high. This indicates that the anthocyanin content can be predicted accurately.

SG, SNV, DET, and their combinations were used to pretreat the original spectrum of the endothelium. The SG + DET + CNN model exhibited the best prediction performance, with Rc = 0.9499, RMSEC = 0.0359%, Rp = 0.9439, RMSEP = 0.2384%, and RPD = 4.6516. The RPD value of this model was also very high, indicating that it is very robust and can accurately predict anthocyanin content.

The CARS and SPA algorithms were used to extract feature wavelength variables. The DET + CARS + CNN model exhibited the best prediction performance: Rc = 0.9527, RMSEC = 0.0708%, Rp = 0.9457, RMSEP = 0.2711%, and RPD = 4.1623. The RPD value of this model is also very high. However, the prediction accuracy of this model was worse than that of the Raw + CNN and SG + DET + CNN models, indicating that the extracted feature variable information was incomplete and inaccurate. The research also found that the prediction accuracy of the CNN model based on SPA is worse than that of the CNN model based on CARS, owing to the small input variable information of SPA and the small amount of feature information learned in the training process of the convolutional neural network. In addition, the characteristic variable information extracted by the SPA in this study was not accurate, which led to a decline in the model prediction accuracy.

**Figure 7:**
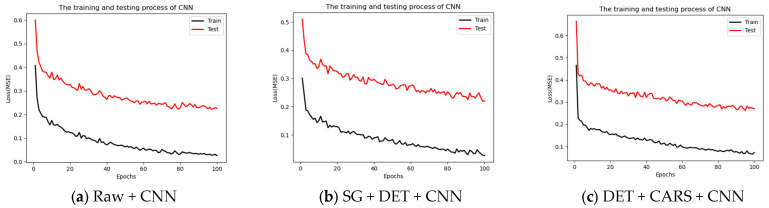
Results of the optimal inner skin model.

In the CNN model constructed by the inner skin part, the training and testing processes of Raw + CNN, SG + DET + CNN, DET + CARS + CNN are shown in Figure 7.

By comparing Table 6 and Table 7, the CNN prediction model of potato anthocyanin content built using different pretreatment methods and feature extraction algorithms for both the skin and endothelium showed little change in the correlation coefficient and root-mean-square error of the model. Compared with the CNN prediction model based on pre-processing and feature extraction algorithms, the CNN model based on the original full spectrum has a better effect, which is basically consistent with the conclusions of previous studies. Zhang Xiaolei [23] used a convolutional neural network to carry out end-to-end qualitative analysis of the spectral data of grape varieties and found that the classification and recognition effect without pretreatment was better. At the same time, the protein content of corn, the active substance content of tablets, the protein content of wheat, and the organic carbon content of soil are also quantitatively analyzed, and it is concluded that the convolutional neural network can learn from the original data without pretreatment, thus improving the accuracy.

LeCun Y [23] uses traditional algorithms and deep learning convolutional neural network algorithms to identify the feature information in the image. It is found that convolutional neural network algorithms realize linear and nonlinear expression, and identify image features adaptively under the action of different network layers and inverse functions, which reduces the pre-processing links of conventional algorithms.

Xin Wang [44] proposed an end-to-end deep learning method called the residual spectrum, which combined residual modules to learn features from original data to improve model performance and compared this algorithm with the classical convolutional neural network. The results show that the residual spectrum is better than other CNN models and traditional machine learning models in the original data.

To a certain extent, deep learning has the ability to automatically extract feature information, and extracting spectral features does not require manual pretreatment. This also shows that the CNN algorithm can simplify several operation processes of traditional machine learning algorithms and effectively improve the efficiency of model prediction.

In addition, the results showed that the accuracy of the CNN prediction model established by the inner skin part was better than that established by the outer skin part, which is consistent with the prediction model established by the previous PLS algorithm.

## 4. Conclusions

Based on a basic convolutional neural network, this study proposes the use of normalization and activation functions to build a network framework for processing spectral data information. The optimized CNN network structure and traditional machine learning PLS algorithm were used to construct potato internal anthocyanin level prediction models, and the best algorithm was selected for comparative analysis.

CNN and PLS models for anthocyanin content prediction were constructed based on the original spectrum. Rp = 0.7765, RMSEP = 0.426%, Rc = 0.7874, RMSEC = 0.429%, RPD = 2.30 for the Raw + PLS prediction model constructed in the outer skin. Rp = 0.8429, RMSEP = 0.403%, Rc = 0.8159, RMSEC = 0.422%, RPD = 2.45 for the Raw + PLS prediction model constructed in the inner skin part. Rc = 0.9486, RMSEC = 0.0312%, Rp = 0.9446, RMSEP = 0.2291%, RPD = 4.41 for the Raw + CNN prediction model constructed in the outer skin. Rc = 0.9508, RMSEC = 0.0374%, Rp = 0.9461, RMSEP = 0.2361%, and RPD = 4.4933 for the Raw + CNN prediction models constructed for the inner skin part. The results showed that the accuracy of the prediction model established by the CNN algorithm was better than that of the conventional machine learning algorithm PLS model, and the prediction accuracy of the model established using the spectral information of the endothelium was better than that of the model established using the spectral information of the outer skin.

SG, SNV, SET, SG + SNV, SG + DET, SNV + DET, and other algorithms were used for the pre-processing of the original spectrum. After pre-processing, the PLS prediction model and CNN prediction models for anthocyanin content were constructed. The specific results are as follows: for the prediction model built based on PLS, the SNV + PLS algorithm was the best model for the potato skin (Rp = 0.7496, RMSEP = 0.435%, Rc = 0.7439, RMSEC = 0.449%, and RPD = 2.25). For the inner skin part, the SNV + PLS algorithm established the best model, with Rp = 0.839, RMSEP = 0.395%, Rc = 0.7707, RMSEC = 0.446%, and RPD = 2.50. In the model constructed based on the CNN, the DET + CNN model was the best for the potato skin, with Rc = 0.9499, RMSEC = 0.0235%, Rp = 0.9457, RMSEP = 0.2234%, and RPD = 4.4893; in the inner skin part, the SG + DET + CNN model was the best, with Rc = 0.9499 and RPD = 4.4893, RMSEC = 0.0359%, Rp = 0.9439, RMSEP = 0.2384%, and RPD = 4.6516. From the above data, it can be concluded that the CNN algorithm established using deep learning is superior to the conventional PLS algorithm and it shows that the prediction model established by the inner skin part is better than that by the outer skin part of the potato.

For the pre-processed spectral data, the CARS and SPA algorithms were used to select the variables most related to the anthocyanin content, and then the PLS and CNN prediction models were established. The specific results were as follows: for the model built based on PLS, DET + CARS + PLS was the best model for the skin (Rp = 0.8593, RMSEP = 0.37%, Rc = 0.8158, RMSEC = 0.334%, and RPD = 2.64). For inner skin cells, DET + CARS + PLS was the best model (Rp = 0.9287, RMSEP = 0.325%, Rc = 0.9227, RMSEC = 0.329%, and RPD = 3.03). In the model constructed based on the CNN, DET + CARS + CNN was the best model for the skin (Rc = 0.9524, RMSEC = 0.0657%, Rp = 0.9468, RMSEP = 0.2651%, RPD = 4.0942), and DET + CARS + CNN was the best model (Rc = 0.9527, RMSEC = 0.0708%, Rp = 0.9457, RMSEP = 0.2711%, and RPD = 4.1623). According to the above data, the CNN neural network algorithm is more ideal than the PLS algorithm for the model established by the feature variable extraction method, and the model built in the inner skin part is better than the model built in the outer skin part.

According to the data, the models established based on the feature variables were worse than those established based on the original spectrum and pre-processing. This was because the data extracted from the feature variables contained less information than the original and pre-processed spectral information. Compared with the CNN algorithm, the models constructed using pre-processing and feature variable extraction showed little difference in correlation coefficients and RMS errors. These results provide meaningful directions for building deep learning models using spectral information in the future. The results also showed that the sampling position affected the prediction accuracy of the model, providing a reference for improving the prediction effect of the model in the future.

## Figures and Tables

**Table 1:** Parameters of potato micro-hyperspectral image acquisition.

System Parameter	Parameter Value
Wavelength range	365–1025 nm
Number of bands	616
Image space size	816 × 616
Objective factor	20
Moving speed	0.2 mm/s
Exposure time	100 ms

Instrument parameters are default values.

**Table 2:** Characteristic wavelength information of the outer skin.

Method	All-Band Number	Number of Characteristic Variables	Specific Band
SG + CARS	616	47	370,377,378,378,535,560,579,606,636,661, 763,770,781,782,783,814,819,821,827,842, 845,856,866,880,889,893,905,907,916,921, 927,947,949,969,973,978,987,993,1003, 1004,1008,1019,1020,1021,1023,1024
SNV + CARS	616	59	365,372,373,378,383,392,396,398,419,425, 457,494,534,535,574,596,648,654,661,688, 690,700,745,750,784,813,814,818,820,821, 824,825,827,828,837,843,849,850,879,886, 889,892,894,900,905,906,916,935,942,950, 951,957,964,970,977,979,989,1008,1023
DET + CARS	616	75	365,370,373,378,378,383,395,398,409,418, 446,473,494,541,542,552,566,574,648,649, 660,688,699,736,780,783,796,807,809,814, 818,819,827,831,837,841,843,847,849,864, 874,879,881,886,889,894,897,904,906,907, 915,916,917,922,928,946,949,950,951,953, 957,966,967,969,977,997,1001,1003,1004, 1005,1008,1010,1020,1023,1024
SG + SNV + CARS	616	42	365,374,378,379,384,408,419,438,479,493, 534,535,551,552,560,662,689,699,750,783, 813,814,819,821,825,827,836,845,848,866, 872,880,893,906,923,947,970,988,1004,1019,1020,1023
SG + DET + CARS	616	67	370,373,375,376,378,378,379,409,417,495, 534,535,560,561,565,592,602,660,690,764, 781,782,792,806,813,814,819,821,827,836, 842,843,845,848,864,866,879,880,889,890, 893,905,906,907,914,922,923,931,932,947, 948,949,965,968,969,978,988,1003,1004, 1005,1008,1019,1020,1021,1023,1024
SNV + DET + CARS	616	53	373,378,391,420,489,494,535,560,575,662, 691,699,712,750,781,784,807,813,814,816, 818,820,824,825,827,837,843,857,867,873, 874,883,886,889,892,894,897,901,906,907, 920,922,928,932,938,946,947,950,951,953,977,1005,1020
**SG + SPA**	616	12	379,381,696,820,845,857,880,931,947,1003,1021,1023
SNV + SPA	616	29	383,388,390,418,465,489,541,646,663,685, 706,712,763,802,809,813,816,818,819,823, 824,825,826,846,849,861,867,879,1017
DET + SPA	616	19	480,624,637,664,686,706,827,890,951,953, 957,964,965,970,976,978,988,1003,1010
SG + SNV + SPA	616	53	365,372,378,382,408,423,486,573,577,596, 612,621,624,641,653,662,665,688,697,706, 712,729,734,739,742,750,778,794,798,814, 816,818,820,827,837,845,853,866,869,872, 875,888,893,907,926,929,940,947,958,964, 967,994,1021
SG + DET + SPA	616	63	408,423,451,492,585,617,619,625,629,633, 635,659,670,674,677,693,704,710,715,730, 818,827,831,835,838,848,878,899,907,911, 916,918,921,923,925,931,936,944,946,948, 949,951,954,955,956,966,967,968,970,978, 980,981,989,992,994,996,1002,1004,1008, 1019,1021,1023
SNV + DET + SPA	616	12	636,670,677,708,852,881,951,957,964,968, 989,990

The algorithms that are shown individually in bold in the table are the ones that are the focus of the following analysis.

**Table 3:** Characteristic wavelength information of the inner skin.

Method	All-Band Number	Number of Characteristic Variables	Specific Band
SG + CARS	616	59	378,484,524,525,546,549,558,562,570, 582,585,590,608,625,632,635,637,683, 748,756,772,775,792,793,796,814,827, 828,835,854,857,862,866,873,874,889, 893,896,902,905,907,912,914,915,921, 935,940,956,962,970,971,988,1004, 1014,1016,1021,1023,1024
SNV + CARS	616	53	368,380,389,392,409,422,448,507,570, 571,626,646,655,685,755,766,773,777, 782,789,792,793,798,812,814,818,827, 832,837,851,853,855,889,891,897,903, 905,907,926,934,951,957,967,972,974, 987,989,994,1010,1020,1022,1023,1025
DET + CARS	616	84	369,370,378,388,389,422,426,519,548, 553,570,571,582,583,586,590,625,626, 627,630,632,635,637,668,683,693,739, 753,754,755,765,766,773,782,784,789, 793,796,798,810,814,819,827,837,855, 856,859,865,874,883,889,891,895,897, 902,905,906,907,916,921,922,926,927, 934,935,940,942,949,953,957,962,967, 970,971,974,975,989,994,1007,1014, 1017,1022,1023,1025
SG + SNV + CARS	616	59	378,379,390,397,405,507,512,558,563, 570,571,643,655,685,752,766,771,773, 776,777,792,793,797,812,813,814,826, 827,829,856,862,866,873,875,887,889, 894,898,906,915,923,930,941,946,956, 959,971,972,980,988,989,993,994, 1005,1014,1017,1019,1021,1025
SG + DET + CARS	616	42	507,518,520,526,548,549,570,590,594, 599,625,630,740,754,767,782,788,793, 797,805,813,827,856,865,875,889,894, 902,907,912,914,922,940,956,959,961, 970,988,1004, 1021,1023,1024
SNV + DET + CARS	616	37	408,505,559,562,565,571,590,630,752, 764,771,792,794,795,815,827,830,832, 835,840,850,859,860,889,891,892,904, 906,957,966,967,985,989,993,1022,1023,1024
SG + SPA	616	20	367,537,577,654,681,716,830,856,859, 907,914,921,934,957,959,960,987,988, 1020,1021
SNV + SPA	616	9	391,588,636,704,793,813,826,867,873
DET + SPA	616	12	625,685,821,854,891,946,947,971,976, 989,993,1016
SG + SNV + SPA	616	33	379,382,400,577,590,593,618,636,639, 643,646,651,654,678,681,729,734,751, 760,802,814,820,824,827,835,868,871, 874,923,929,947,992,1014
SG + DET + SPA	616	31	365,389,460,470,611,619,625,629,636, 638,670,683,710,814,824,828,874,907, 922,929,940,949,956,959,960,969,971, 981,1002,1004,1020
**SNV + DET + SPA**	616	11	533,854,939,946,950,957,961,965,989, 993,998

The algorithms that are shown individually in bold in the table are the ones that are the focus of the following analysis.

**Table 4:** Regression prediction of PLS in outer skin region.

Treatment Method	Rp	RMSEP	Rc	RMSEC	RPD
Primary spectrum (Raw)	0.7765	0.426	0.7874	0.429	2.30
SG	0.7161	0.448	0.7216	0.459	2.18
**SNV**	0.7496	0.435	0.7439	0.449	2.25
DET	0.7446	0.444	0.7928	0.425	2.20
SG + SNV	0.7237	0.451	0.7249	0.462	2.17
SG + DET	0.7130	0.457	0.7227	0.469	2.14
SNV + DET	0.7419	0.437	0.7490	0.444	2.24
SG + CARS	0.8267	0.387	0.8509	0.380	2.53
SNV + CARS	0.8462	0.373	0.8410	0.386	2.62
**DET + CARS**	0.8593	0.370	0.8158	0.334	2.64
SG + SNV + CARS	0.8382	0.378	0.8147	0.403	2.59
SG + DET + CARS	0.8398	0.378	0.8429	0.386	2.59
SNV + DET + CARS	0.8409	0.378	0.8359	0.389	2.59
SG + SPA	0.7464	0.428	0.7837	0.466	2.29
SNV + SPA	0.7123	0.445	0.7274	0.448	2.20
DET + SPA	0.6316	0.477	0.6233	0.491	2.05
SG + SNV + SPA	0.7347	0.433	0.7093	0.455	2.26
SG + DET + SPA	0.7263	0.454	0.7897	0.418	2.15
SNV + DET + SPA	0.6281	0.477	0.6046	0.495	2.05

The algorithms that are shown individually in bold in the table are the ones that are the focus of the following analysis.

**Table 5:** Regression prediction of PLS in inner skin region.

Treatment Method	Rp	RMSEP	Rc	RMSEC	RPD
Primary spectrum (Raw)	0.8429	0.403	0.8159	0.422	2.45
SG	0.8205	0.421	0.7877	0.441	2.35
**SNV**	0.8390	0.395	0.7707	0.446	2.50
DET	0.8334	0.406	0.7985	0.431	2.43
SG + SNV	0.8397	0.396	0.7509	0.452	2.50
SG + DET	0.7870	0.429	0.7814	0.439	2.30
SNV + DET	0.8370	0.397	0.7566	0.449	2.49
SG + CARS	0.9086	0.346	0.9019	0.346	2.85
SNV + CARS	0.8868	0.360	0.8524	0.381	2.74
**DET + CARS**	0.9287	0.325	0.9227	0.329	3.03
SG + SNV + CARS	0.8832	0.362	0.8577	0.383	2.73
SG + DET + CARS	0.9178	0.332	0.8738	0.371	2.97
SNV + DET + CARS	0.9057	0.345	0.8561	0.378	2.86
SG + SPA	0.8668	0.376	0.8134	0.413	2.63
SNV + SPA	0.7829	0.432	0.7145	0.467	2.29
DET + SPA	0.8123	0.414	0.7501	0.450	2.38
SG + SNV + SPA	0.8087	0.417	0.7870	0.434	2.37
SG + DET + SPA	0.8139	0.414	0.8001	0.422	2.38
SNV + DET + SPA	0.8220	0.410	0.7708	0.439	2.41

The algorithms that are shown individually in bold in the table are the ones that are the focus of the following analysis.

**Table 6:** CNN-anthocyanin prediction model in outer skin.

Treatment Method	Input Variable	Rc	RMSEC	Rp	RMSEP	RPD
Primary spectrum (Raw)	616	0.9486	0.0312	0.9446	0.2291	4.41
SG	616	0.9506	0.0312	0.9440	0.2339	4.3728
SNV	616	0.9503	0.0274	0.9446	0.2273	4.4556
**DET**	616	0.9499	0.0235	0.9457	0.2234	4.4893
SG + SNV	616	0.9487	0.0318	0.9452	0.2309	4.4367
SG + DET	616	0.9518	0.0278	0.9445	0.2282	4.4782
SNV + DET	616	0.9504	0.0225	0.9456	0.2237	4.3895
SG + CARS	47	0.9539	0.1103	0.9494	0.3137	3.7346
SNV + CARS	59	0.9527	0.0815	0.9501	0.2843	3.9260
**DET + CARS**	75	0.9524	0.0657	0.9468	0.2651	4.0942
SG + SNV + CARS	42	0.9536	0.1028	0.9483	0.300	3.8091
SG + DET + CARS	67	0.9509	0.0673	0.9474	0.2687	4.0421
SNV + DET + CARS	53	0.9549	0.0909	0.9484	0.2911	3.9047
SG + SPA	12	0.9600	0.2019	0.9543	0.4012	3.3207
SNV + SPA	29	0.9565	0.1375	0.9519	0.3379	3.6080
DET + SPA	19	0.9573	0.1823	0.9526	0.3839	3.4089
SG + SNV + SPA	53	0.9524	0.0776	0.9492	0.2767	3.9991
SG + DET + SPA	63	0.9536	0.0857	0.9479	0.2860	3.9538
SNV + DET + SPA	12	0.9592	0.2205	0.9541	0.4232	3.2656

The algorithms that are shown individually in bold in the table are the ones that are the focus of the following analysis.

**Table 7:** CNN-anthocyanin prediction model in inner skin.

Treatment Method	Input Variable	Rc	RMSEC	Rp	RMSEP	RPD
Primary spectrum (Raw)	616	0.9508	0.0374	0.9461	0.2361	4.4933
SG	616	0.9518	0.0356	0.9444	0.2364	4.5894
SNV	616	0.9484	0.0380	0.9450	0.2390	4.5236
DET	616	0.9512	0.0354	0.9450	0.2348	4.5869
SG + SNV	616	0.9501	0.0392	0.9458	0.2356	4.5635
**SG + DET**	616	0.9499	0.0359	0.9439	0.2384	4.6516
SNV + DET	616	0.9509	0.0364	0.9453	0.2402	4.4951
SG + CARS	59	0.9526	0.0891	0.9489	0.2876	4.1448
SNV + CARS	53	0.9540	0.0998	0.9487	0.3001	4.0022
**DET + CARS**	84	0.9527	0.0708	0.9457	0.2711	4.1623
SG + SNV + CARS	59	0.9530	0.0988	0.9476	0.2997	4.0345
SG + DET + CARS	42	0.9542	0.1201	0.9512	0.3118	3.8870
SNV + DET + CARS	37	0.9542	0.1168	0.9473	0.3194	3.9253
SG + SPA	20	0.9565	0.1419	0.9510	0.3409	3.8222
SNV + SPA	9	0.9596	0.2092	0.9559	0.4103	3.4881
DET + SPA	12	0.9561	0.1649	0.9511	0.3672	3.6420
SG + SNV + SPA	33	0.9521	0.0995	0.9479	0.3010	4.0386
SG + DET + SPA	31	0.9542	0.1210	0.9492	0.3221	3.8943
SNV + DET + SPA	11	0.9592	0.2184	0.9534	0.4201	3.3842

The algorithms that are shown individually in bold in the table are the ones that are the focus of the following analysis.

## Data Availability

The data presented in this study are available on request from the corresponding author.
